# Supplementary material for: Kif2 localizes to a subdomain of cortical endoplasmic reticulum that drives asymmetric spindle position
Source: Nat Commun. 2017 Oct 13;8:917. doi: 10.1038/s41467-017-01048-8 (PMC5640700; doi:10.1038/s41467-017-01048-8)
Supplement: Supplementary file 1 — Supplementary Information [file 41467_2017_1048_MOESM1_ESM.pdf]

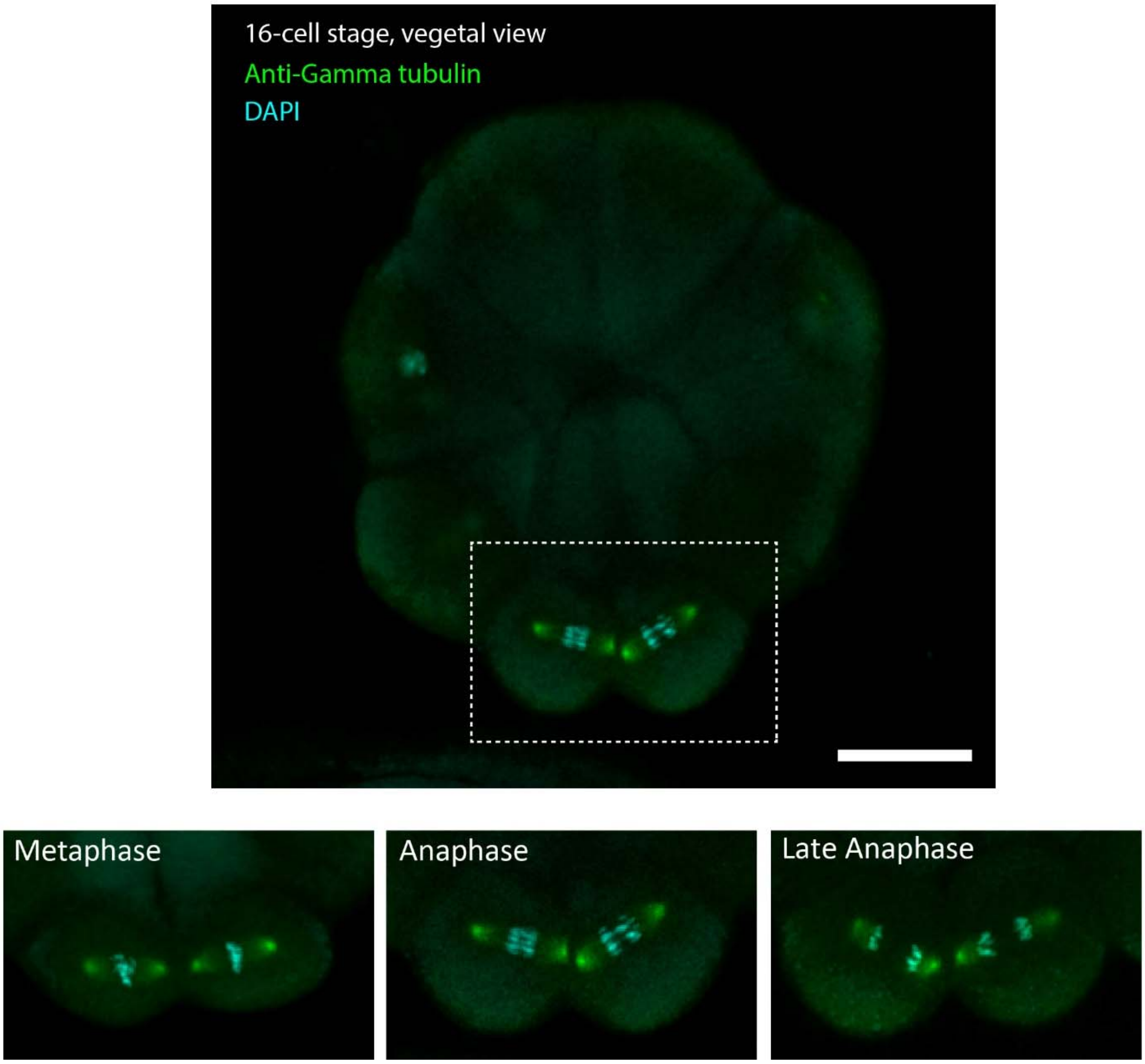

Supplementary Figure 1. Gamma-tubulin presence at spindle poles during mitosis.

16-cell stage *Phallusia mammillata* embryos fixed and labelled with anti-γ-tubulin (green) antibody and DAPI (blue). Inset showing γ-tubulin at metaphase, anaphase and late anaphase of the posterior pair of blastomeres (B5.2). γ-tubulin is present at both spindle poles throughout mitosis. Scale bar = 30µm.

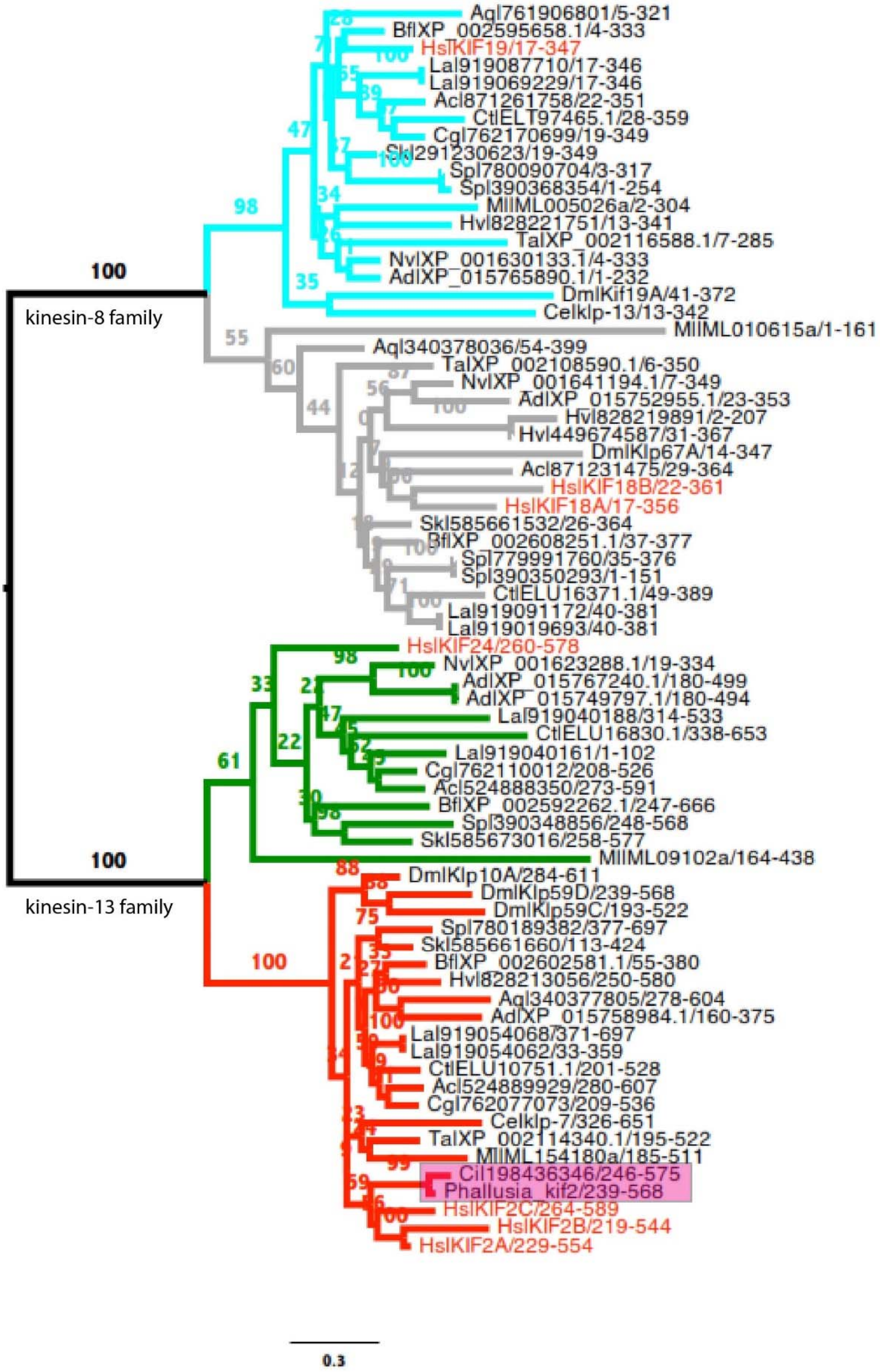
Aql761906801/5-321
BflXP_002595658.1/4-333
HslKIF19/17-347
Lal919087710/17-346
Lal919069229/17-346
Acl871261758/22-351
CtlELT97465.1/28-359
Cgl762170699/19-349
Skl291230623/19-349
Spl780090704/3-317
Spl390368354/1-254
MlIML005026a/2-304
Hvl828221751/13-341
TalXP_002116588.1/7-285
NvlXP_001630133.1/4-333
AdlXP_015765890.1/1-232
DmlKif19A/41-372
Celklp-13/13-342
MlIML010615a/1-161
Aql340378036/54-399
TalXP_002108590.1/6-350
NvlXP_001641194.1/7-349
AdlXP_015752955.1/23-353
Hvl828219891/2-207
Hvl449674587/31-367
DmlKlp67A/14-347
Acl871231475/29-364
HslKIF18B/22-361
HslKIF18A/17-356
Skl585661532/26-364
BflXP_002608251.1/37-377
Spl779991760/35-376
Spl390350293/1-151
CtlELU16371.1/49-389
Lal919091172/40-381
Lal919019693/40-381
HslKIF24/260-578
NvlXP_001623288.1/19-334
AdlXP_015767240.1/180-499
AdlXP_015749797.1/180-494
Lal919040188/314-533
CtlELU16830.1/338-653
Lal919040161/1-102
Cgl762110012/208-526
Acl524888350/273-591
BflXP_002592262.1/247-666
Spl390348856/248-568
Skl585673016/258-577
MlIML09102a/164-438
DmlKlp10A/284-611
DmlKlp59D/239-568
DmlKlp59C/193-522
Spl780189382/377-697
Skl585661660/113-424
BflXP_002602581.1/55-380
Hvl828213056/250-580
Aql340377805/278-604
AdlXP_015758984.1/160-375
Lal919054068/371-697
Lal919054062/33-359
CtlELU10751.1/201-528
Acl524889929/280-607
Cgl762077073/209-536
Celklp-7/326-651
TalXP_002114340.1/195-522
MlIML154180a/185-511
Cil198436346/246-575
Phallusia_kif2/239-568
HslKIF2C/264-589
HslKIF2B/219-544
HslKIF2A/229-554
100
kinesin-8 family
100
kinesin-13 family
98
55
61
100
0.3

Supplementary Figure 2. Phylogenetic analysis of Kif2 (kinesin-13) family of proteins.

Phyml tree of the clade of animal KIF19, KIF18, KIF24 and KIF2A/B/C ortholog groups. Kif2 and Kif24 (bottom half of tree) are members of the Kinesin-13 family of microtubule depolymerases; Kif18 and Kif19 (top half of tree) are members of the related Kinesin-8 family [16]. With the exception of the *Phallusia* sequence, the first two letters of leaf names represent the species of the sequence (see methods for list). These are followed by identifiers retrievable from the NCBI protein sequence database. Bootstrap support is show above branches. Although within the gene groups the species phylogeny is generally not well resolved, there is high support for lineage specific duplications of Kif2 in *Drosophila* and Humans, with the majority of invertebrate taxa having only a single representative, strongly suggesting the state in the ancestral animal was a single gene. *Ciona* and *Phallusia* data are highlighted by the pink box.

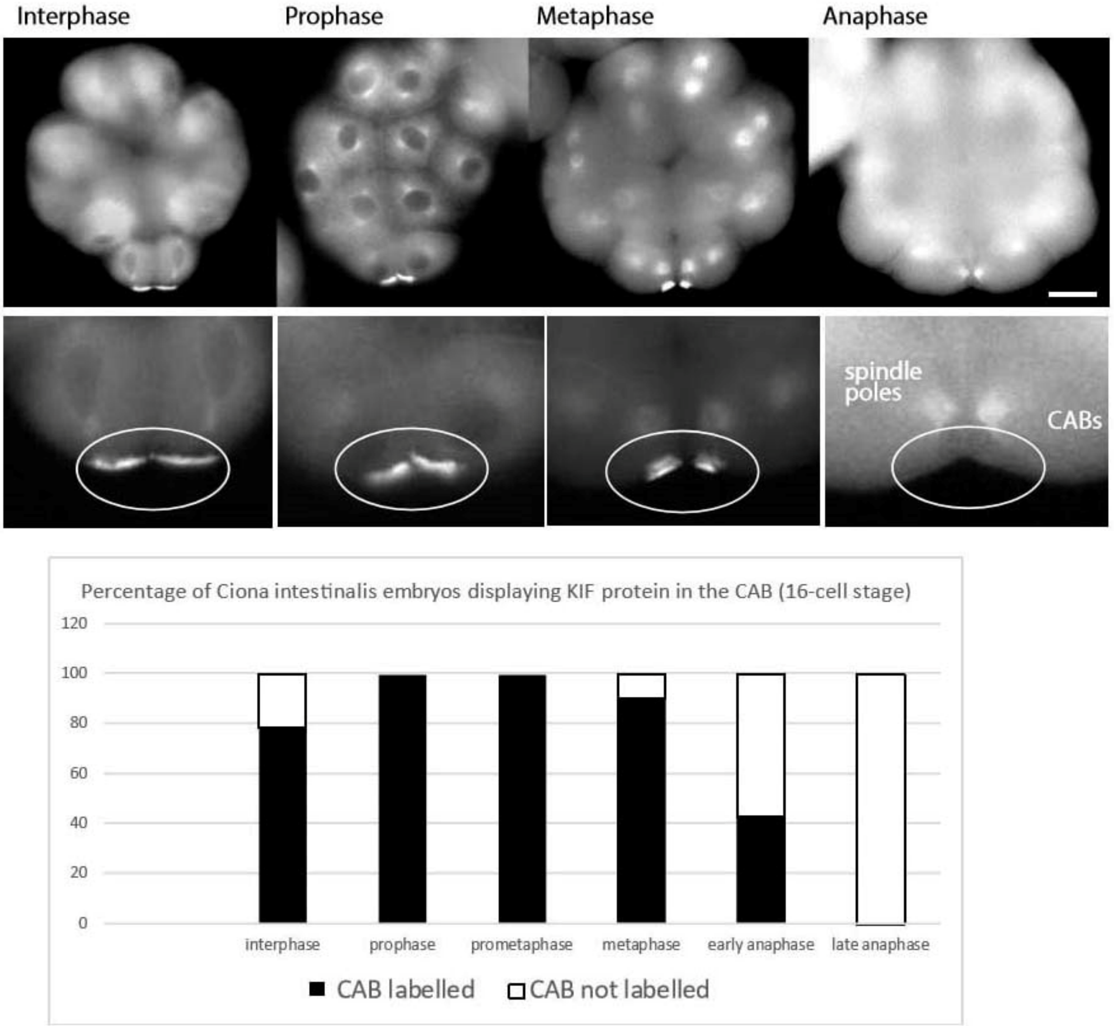

Supplementary Figure 3. Endogenous Kif2 protein localization at the CAB in *Ciona intestinalis*.

Immunolabelling with anti-Kif2 of 16-cell stage *Ciona* embryos. Kif2 labels the CAB during later interphase/early prophase and is absent at the CAB during anaphase. Scale bar = 30 µm. Graph showing percentage of embryos (total n=94) with CAB labelled at different stages of the cell cycle. Note, these percentages do not indicate amount of Kif2 at the CAB but presence or absence.

Control sperm aster

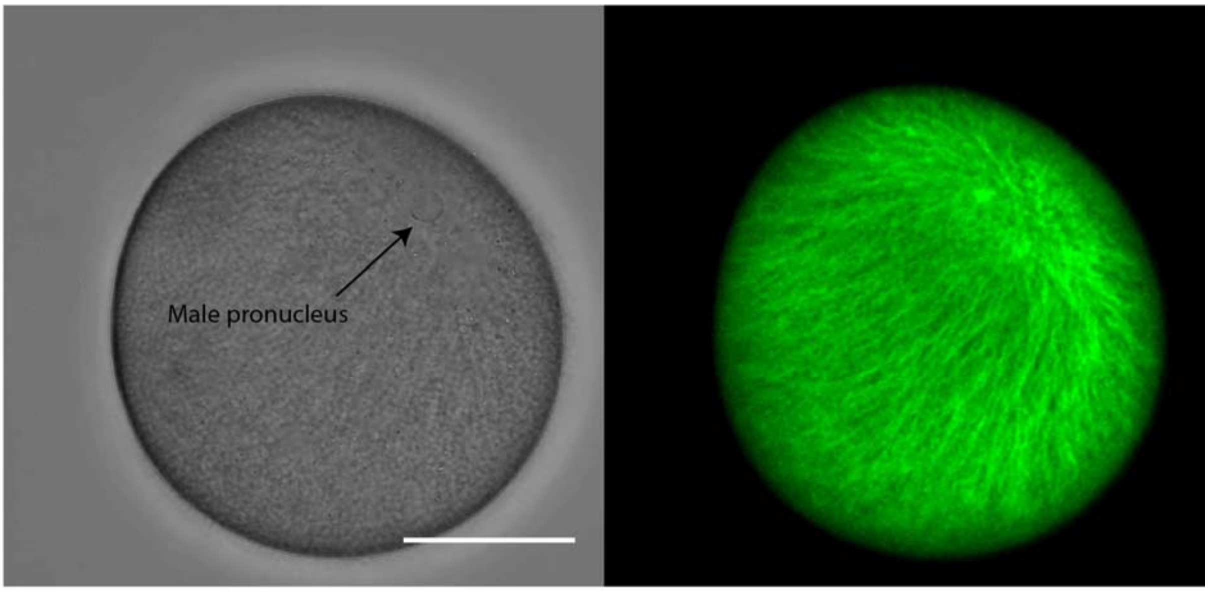

Sperm aster in the presence of Kif2 overexpression

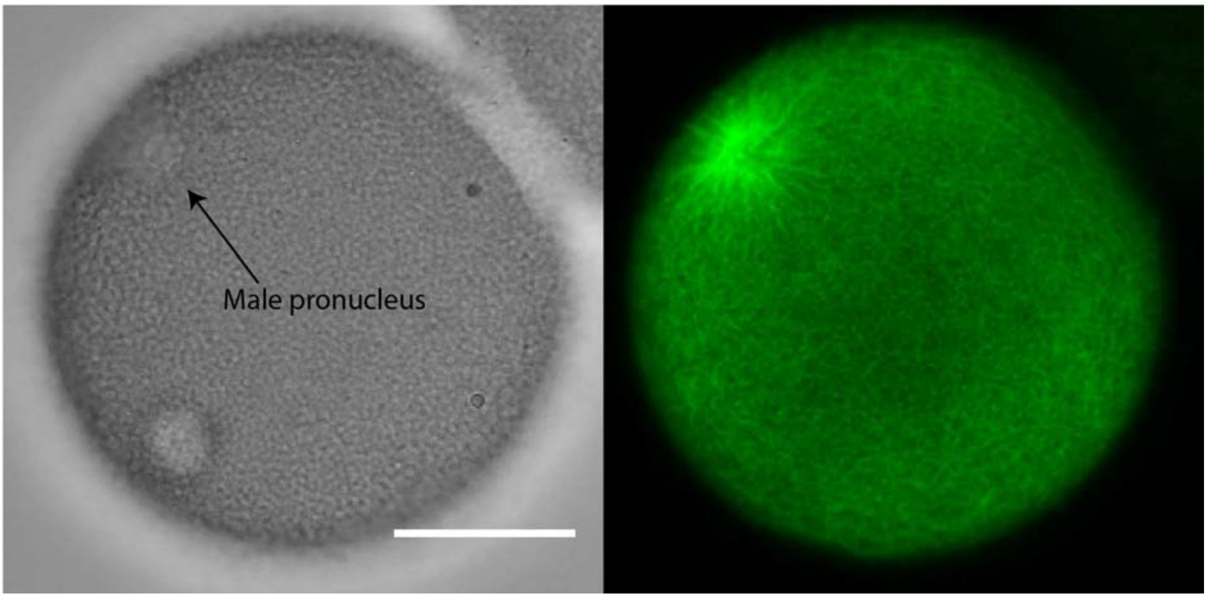

Supplementary Figure 4. Microtubule density is reduced in cells overexpressing Kif2.

Eggs injected with mRNAs encoding Ens::3GFP (top pair of images) or Kif2 (without a fluorescent tag) plus Ens::3GFP (bottom pair of images) and were left overnight then fertilized. Confocal images from a z stack showing that in eggs overexpressing Kif2 microtubule density of the sperm aster was greatly reduced (bottom fluorescence image) compared to a zygote without exogenous Kif2 (upper fluorescence image). Bright-field images showing the presence of the male pronucleus (arrows) in both zygotes. Scale bars = 50µm. See Supplementary Movie 14.

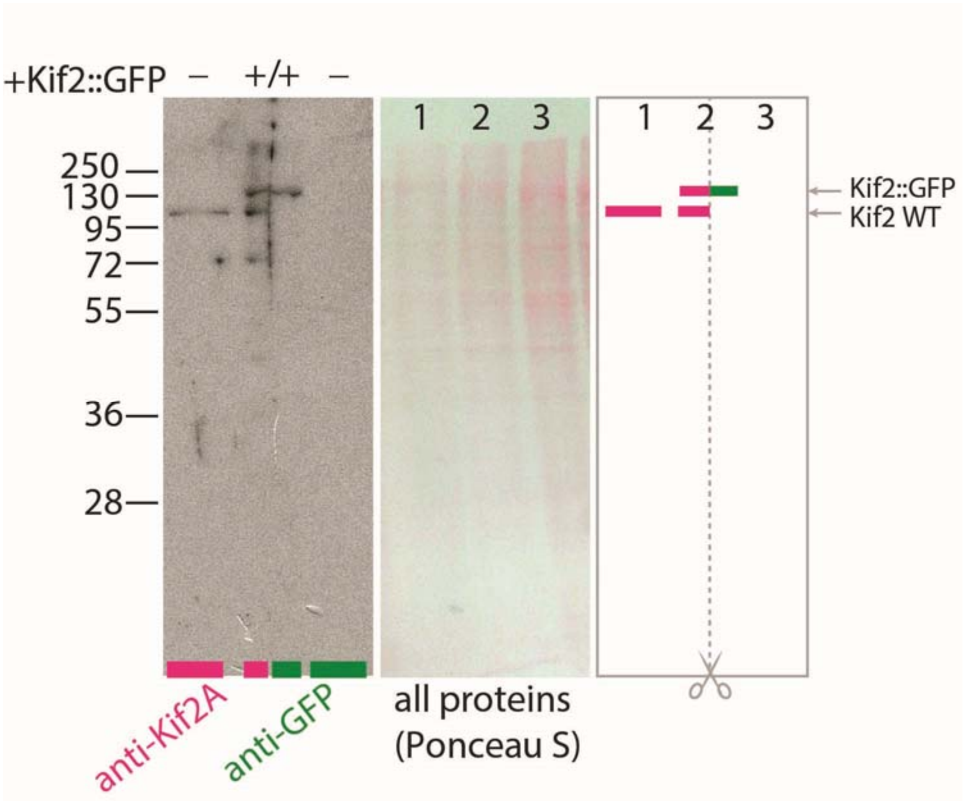

Supplementary Figure 5. Western blot of Kif2.

Western blot showing that Kif2 protein is present in unfertilized eggs. Extracts of unfertilized *Phallusia* eggs that were either uninjected (-) or injected (+) with mRNA encoding Kif2::GFP and probed with anti-Kif2 or anti-GFP (left), Ponceau S showing proteins (center) and schematic showing labelling pattern (right). Green bar represents the Kif2::GFP which migrates at a higher molecular mass than endogenous Kif2 protein (pink bar). Some degradation product can be seen in the anti-Kif2A lane.

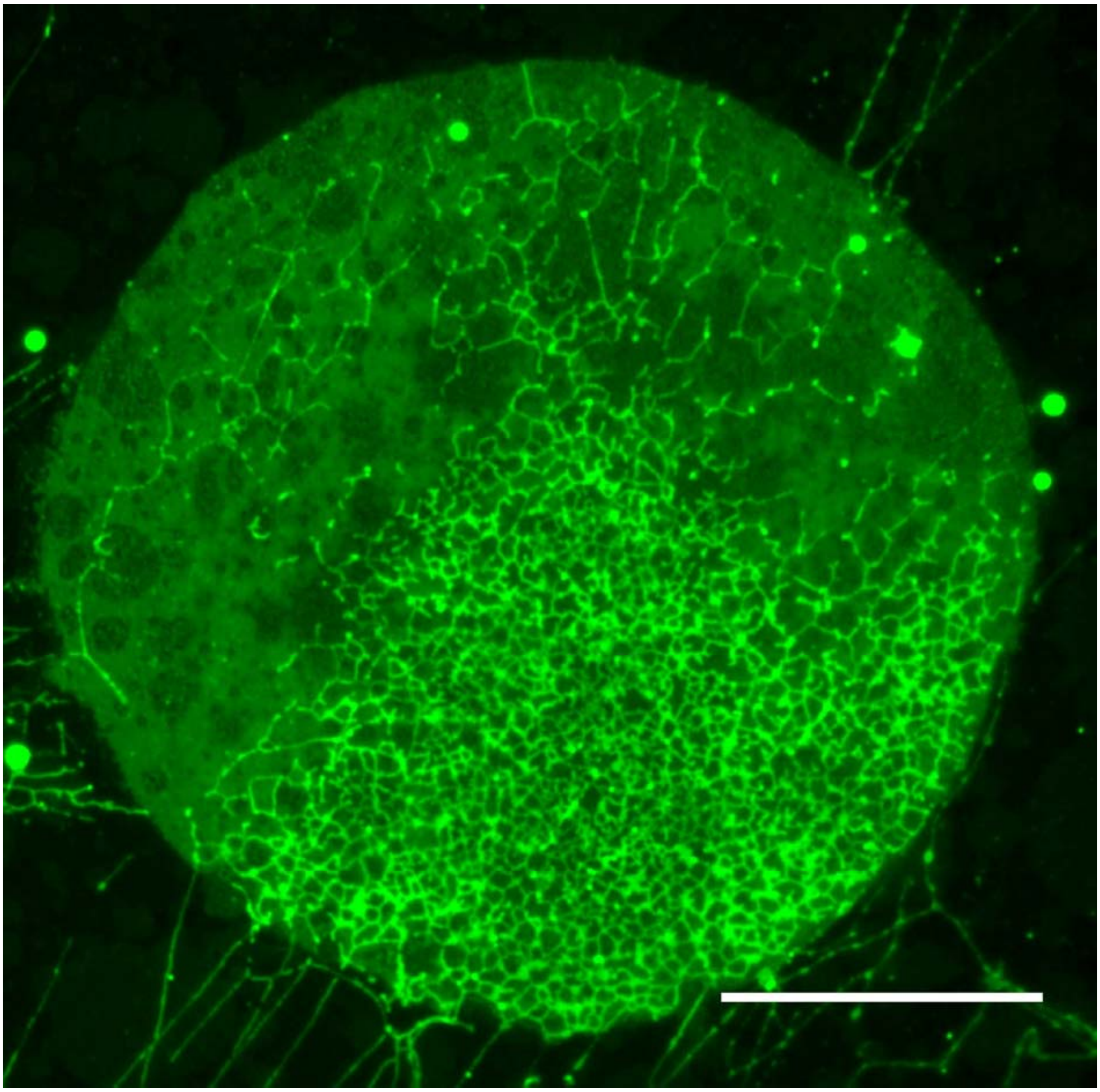

Supplementary Figure 6. Cortical preparation of DiO-labelled cortical endoplasmic reticulum in an egg.

Cortical preparations of unfertilized eggs were prepared as indicated in the material section for embryos. DiO labelling shows the reticular structure typical of the cortical ER of unfertilized eggs. Scale bar = 20 µm.

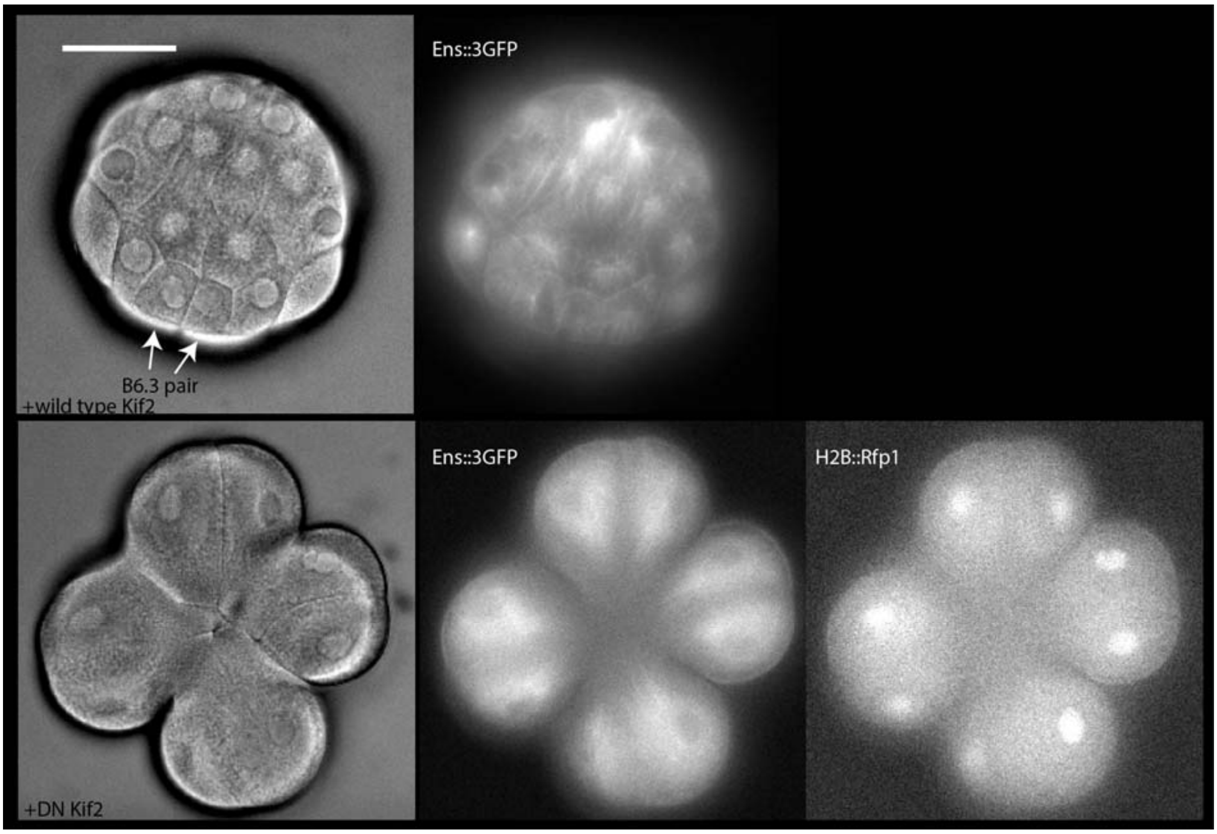

Supplementary Figure 7. Wild type versus dominant negative Kif2.

Unfertilized eggs were microinjected with mRNA encoding wild type Kif2 mixed with Ens::3GFP or dominant negative Kif2 mixed with Ens::3GFP and H2B::mRfp1. The same concentration of mRNA encoding wt Kif2 and DN-Kif2 and Ens::3GFP was used. Bright-field and fluorescence images of embryos containing wt Kif2 or DN Kif2 are shown. None of the DN Kif2-injected eggs reached the 32-cell stage (32/32 embryos scored) while 12/16 wt Kif2 embryos reached the 32-cell stage. Scale bar=50µm.

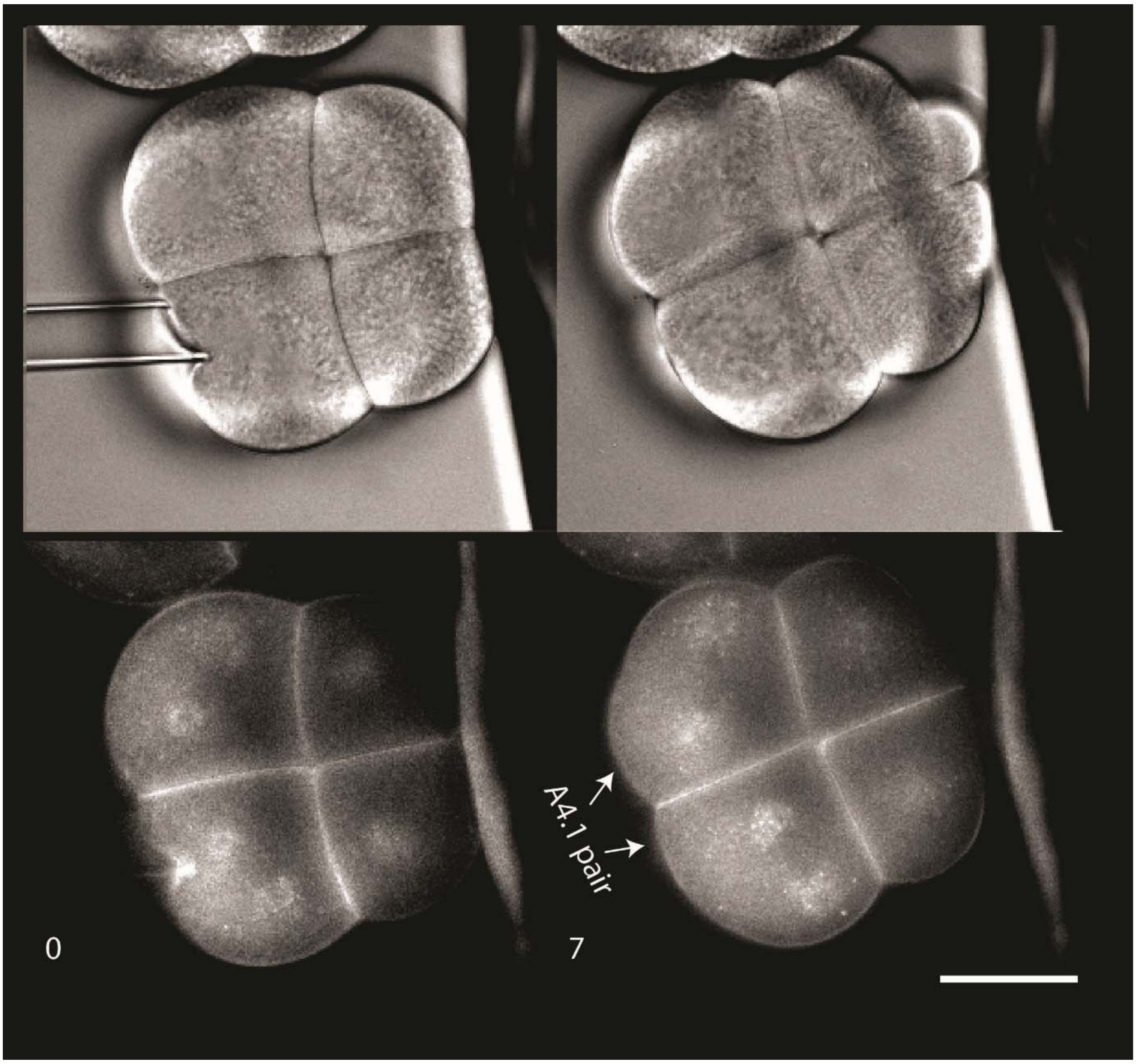

Supplementary Figure 8. Nocodazole pipette applied to non-CAB blastomeres (A4.1).

Embryos at the 8-cell stage (interphase) were bathed in Cell Mask orange to label the mitotic spindle poles and the plasma membrane and during mitosis a nocodazole pipette was applied to the surface of one A4.1 blastomere immediately following NEB. Note the exaggerated movement of the spindle pole nearest the nocodazole pipette away from nocodazole pipette. We measured the spindle pole to midline distance before and after application of the pipette. The distance to the midline before pipette was 19.2+/-2.0 µm and increased to 22.8+/-1.7 µm (mean +/- s.e.m. n=6). Time in min. Scale bar = 50µm.
