# Supplementary material for: Kif2 localizes to a subdomain of cortical endoplasmic reticulum that drives asymmetric spindle position
Source: Nat Commun. 2017 Oct 13;8:917. doi: 10.1038/s41467-017-01048-8 (PMC5640700; doi:10.1038/s41467-017-01048-8)
Supplement: Supplementary file 3 — Description of Additional Supplementary Files [file 41467_2017_1048_MOESM3_ESM.pdf]

Description of Additional Supplementary Files

File Name: Supplementary Movie 1

Description: Unequal cell division from 8 to 16-cell stage.

Bright-field images of 8-cell stage Phallusia embryo viewed from the vegetal side from interphase until cell division. The two vegetal posterior blastomeres that divide unequally (B4.1 pair) are at the bottom. Following nuclear envelope breakdown it is possible to discern the movement of the mitotic spindle in the two bottom blastomeres. The two CAB-containing blastomeres at the 16-cell stage (B5.2 pair) are indicated by the white circles. Time in min.

File Name: Supplementary Movie 2

Description: Unequal Cell Division 1. 8-16-cell

Unfertilized Phallusia eggs injected with Ens::3GFP (green). Confocal images of 8-cell stage embryos showing microtubule behavior during late interphase and mitosis. B4.1 pair of blastomeres are at the bottom. The two CAB-containing blastomeres at the 16-cell stage (B5.2 pair) are indicated by the white circles. Note that the aster is asymmetric in both bottom blastomeres. Time in min. Scale bar = 30µm.

File Name: Supplementary Movie 3

Description: Unequal Cell Division 2. 16 to 32-cell

Unfertilized Phallusia eggs injected with Ens::3GFP (green). Confocal images of 16-cell stage embryo showing microtubule behavior during interphase and mitosis. B5.2 pair of blastomeres are at the bottom. The two CAB-containing blastomeres at the 32-cell stage (B6.3 pair) are indicated by the white circles. Note that the aster becomes asymmetric in both bottom blastomeres. Time in min. Scale bar = 30µm.

File Name: Supplementary Movie 4

Description: Unequal Cell Division 3. 32 to 64-cell

Unfertilized Phallusia eggs injected with Ens::3GFP (green) and also with Kif2::Venus (also appears green) to label the CAB. Confocal images of 32-cell stage embryo showing microtubule behavior during interphase and mitosis. B6.3 pair of blastomeres are at the bottom. The CAB can be seen centered between the two pairs of centrosomes. Note that the upper spindle pole in each B6.3 blastomere approaches the CAB and midline and that the asters nearest the CAB become progressively smaller. The two CAB-containing blastomeres at the 64-cell stage (B7.6 pair) are indicated by the white circles. Time min. Scale bar = 30µm.

File Name: Supplementary Movie 5

Description: 3D rendering of microtubules during UCD

Unfertilized Phallusia eggs injected with Ens::3GFP (green). 3D-rendered confocal z-planes showing astral microtubule behavior merged with bright-field images from an 8-cell stage embryo. Note the size and behavior of the asters nearest the CAB (bottom right blastomeres) versus those far from the CAB in the B4.1 blastomeres. The two CAB-containing blastomeres at the 16-cell stage (B5.2 pair) are indicated by the white circles.

File Name: Supplementary Movie 6

Description: CAB visualization.

Unfertilized Phallusia eggs injected with PH::Tom (red) to label the plasma membrane and also with EB3::GFP (green) to label microtubules. Embryo at the 8-cell stage with the two CAB blastomeres at the bottom. Several z-planes from a confocal time-series rendered in 3D to better visualize the CAB protrusion. Note the CAB can be seen as a protrusion in both blastomeres (red). For clarity, the CABs of each B4.1 blastomere at the 8-cell stage are indicated by the white circles.

File Name: Supplementary Movie 7

Description: Kif2 protein leaves the CAB during mitosis.

Unfertilized eggs injected with EB3::GFP and Kif2::Tom then imaged using a confocal microscope during the 8 to 16-cell stage. Bright-field images are overlayed with the EB3::GFP and Kif2::Tom fluorescence images. Kif2 protein (red) in the CAB can be seen to leave the CAB during mitosis. Scale bar = 30 µm. Time in sec.

File Name: Supplementary Movie 8

Description: Kif2 protein is lost from the CAB after NEB

Selected confocal images from a 4D time series of an 8-cell stage embryo containing Kif2::Tom (red) and Plk1::Ven (green). Two stacks from the 4D time lapse series are shown (Z plane 1 and Z plane 2). Plk1::Ven labels the CAB (Z plane 1, see arrows) and is also a convenient marker of cell cycle phases (see Z plane 2 images) since Plk1::Ven also accumulates in the nucleus during interphase, labels the chromosomes until metaphase then the spindle midzone at anaphase (last image). Following NEB (at 8:50) the Kif2::Tom fluorescence in the CAB falls relative to Plk1::Ven. The Kif2 fluorescence in the CAB has already diminished at 10:36 min. and continued to fall (see 11:29 min. image) relative to the Plk1::Ven fluorescence which stays relatively constant during this time.

File Name: Supplementary Movie 9

Description: Kif2 localizes to cortical endoplasmic reticulum.

Isolated cortices were prepared, fixed and labelled for cortical endoplasmic reticulum (DiO, green) and with anti-Kif2 (red) at the 8-cell stage. Z stacks of confocal optical sections shows the labelling pattern of the cER (green) and Kif2 (red). Note that at the edge of the CAB some tubes of cER can be seen (one is highlighted by the arrow) which are stained homogenously with DiO while the Kif2 labelling is more punctate. Scale bar = 10µm.

File Name: Supplementary Movie 10

Description: Microtubule polymerization around the CAB.

Fast confocal imaging of a cortical z-plane containing the CAB in isolated B5.2 blastomeres. Microtubules labelled with Ens::3GFP (green) and the CAB with Kif2::Tom (red). Note that microtubules polymerize around the CAB and also occasionally in the hole in the center of the CAB. Scale bar = 5µm. Time in sec.

File Name: Supplementary Movie 11

Description: Microtubule growth around the CAB in an intact embryo.

Confocal images of Ens::3GFP (green) and Kif2::Tom (red) fluorescence during interphase of a 32-cell stage embryo. Microtubules can be seen around the CAB (red and labelled with arrow) but they are less abundant in the CAB. Scale bar = 10µm. Time in sec.

File Name: Supplementary Movie 12

Description: Nocodazole pipette to depolymerise CAB proximal microtubules in zygote.

Zygote with microtubules labelled using Ens::3GFP. A nocodazole pipette was advanced towards one aster and led to the loss of microtubules on one side of the zygote. Time in min.

File Name: Supplementary Movie 13

Description: Nocodazole pipette to depolymerize microtubules at the 8-cell stage.

Embryos at the 8-cell stage (interphase) were bathed in Cell Mask orange to label the mitotic spindle poles and the plasma membrane and during mitosis a nocodazole pipette was applied to the surface of one B4.1 blastomere immediately following NEB. Note the exaggerated movement of the spindle pole nearest the nocodazole pipette towards the surface of the blastomere and CAB. Time in min.

File Name: Supplementary Movie 14

Description: Kif2 acts as a microtubule depolymerase.

Unfertilized eggs were microinjected with a mixture of Kif2 and Ens::3GFP mRNA or with Ens::3GFP and left overnight to synthesize protein. The following day the eggs were fertilized. Confocal z-stacks were acquired when the pronuclei formed. Brightfield and fluorescence image stacks are shown for zygotes that contained only Ens::3GFP (upper pair of images) or a mixture of Kif2 and Ens::3GFP (lower pair of images). The sperm aster during interphase occupies the entire zygote in the control but only a portion of the zygote in the presence of Kif2. The male pronuclei are indicate with arrows.
